# Novel insights into the potential applications of stem cells in pulmonary hypertension therapy

**DOI:** 10.1186/s12931-024-02865-4

**Published:** 2024-06-07

**Authors:** Sijia Guo, Dachun Wang^

**Affiliations:** 1 Stem Cell Laboratory, Second Affiliated Hospital of Fujian Medical University, Quanzhou, Fujian China; 2 The Brown Foundation Institute of Molecular Medicine for the prevention of Human Diseases, University of Texas Medical School at Houston, Houston, TX USA

**Keywords:** Pulmonary hypertension, Stem cells, Pulmonary microvascular, Endothelial cells, Alveolar epithelial cells, Potential therapeutic

## Abstract

Pulmonary hypertension (PH) refers to a group of deadly lung diseases characterized by vascular lesions in the microvasculature and a progressive increase in pulmonary vascular resistance. The prevalence of PH has increased over time. Currently, the treatment options available for PH patients have limited efficacy, and none of them can fundamentally reverse pulmonary vascular remodeling. Stem cells represent an ideal seed with proven efficacy in clinical studies focusing on liver, cardiovascular, and nerve diseases. Since the potential therapeutic effect of mesenchymal stem cells (MSCs) on PH was first reported in 2006, many studies have demonstrated the efficacy of stem cells in PH animal models and suggested that stem cells can help slow the deterioration of lung tissue. Existing PH treatment studies basically focus on the paracrine action of stem cells, including protein regulation, exosome pathway, and cell signaling; however, the specific mechanisms have not yet been clarified. Apoptotic and afunctional pulmonary microvascular endothelial cells (PMVECs) and alveolar epithelial cells (AECs) are two fundamental promoters of PH although they have not been extensively studied by researchers. This review mainly focuses on the supportive communication and interaction between PMVECs and AECs as well as the potential restorative effect of stem cells on their injury. In the future, more studies are needed to prove these effects and explore more radical cures for PH.

## Introduction

Pulmonary hypertension (PH) is a serious chronic progressive disease characterized by dysfunction and vascular endothelial injuries of pulmonary arterioles [1, 2], with abnormal obliterative vascular smooth muscle cell proliferation [3], which can further progress to pulmonary vascular remodeling and right ventricular hypertrophy. These lesions may eventually lead to right heart failure and even death. Consistently, PH is defined as a mean pulmonary artery pressure (mPAP) ≥ 25 mmHg observed at right-sided heart catheterization (RHC). The 6th World Symposium on Pulmonary Hypertension proposed a recent hemodynamic definition of PH, mPAP > 20 mmHg, as the upper limit of normal pulmonary artery pressure [4], and the value is associated with a greater risk of mortality, with pulmonary arterial wedge pressure (PAWP) ≤ 15 mm Hg and pulmonary vascular resistance (PVR) ≥ 3 Wood units [5]. Depending on different causes of disease and clinical pathological features, the World Health Organization divides PH into 5 types: pulmonary arterial hypertension, PH due to left heart disease, PH due to lung diseases and/or hypoxia, PH due to pulmonary artery obstruction, and PH with unclear and/or multifactorial mechanisms. The latest hemodynamic definition can help to aid in early PH diagnosis.

The mechanism of PH has not been completely clarified. This disease is closely related to the abnormal structure and function of pulmonary microvessels, mainly including the following points: (1) hypoxia-induced pulmonary arteriolar contraction and thickening of pulmonary arteriolar media smooth muscle; (2) pulmonary inflammatory reaction; and (3) pulmonary microvascular injury and dysfunction. Among these mechanisms, endothelial cells appear to play a particularly important and crucial role. These cells are believed to reverse PH [6]. However, multiple treatments that target a series of endothelial secretions (e.g., endothelin (ET), nitric oxide (NO) and the prostacyclin pathway) and aim to regulate pulmonary vascular endothelial cells and smooth muscle cells have recently been used for PH therapy, thereby controlling the progression of the disease to a certain extent. It is still difficult to fundamentally recover the functions of pulmonary microvasculature and to reverse vascular remodeling, future PH treatment should focus mainly on reversing remodeling of pulmonary vascular diseases.

In recent years, cell therapy has attracted considerable clinical attention for the treatment of various diseases. Increasing studies have shown that cell therapy may provide a novel therapeutic approach to PH. In the treatment of lung disease, there has been constant progress in preclinical animal experiments of stem cell therapy. Numerous studies have provided evidence to demonstrate a potential therapeutic effect of stem cells in acute lung injury (ALI), chronic obstructive pulmonary disease (COPD), acute respiratory distress syndrome (ARDS) and pulmonary fibrosis (PF) [7–10]. To date, PH remains an incurable disease, and cell therapy represents an important future direction for research. As an important platform for cell therapy, stem cells have recently become a widespread focus of study for an increasing number of researchers. This review examines the mechanism underlying the impact of stem cells on reversing vascular remodeling in the treatment of PH from the root.

## PMVEC dysfunction causes PH

Pulmonary microvascular endothelial cells (PMVECs) are thin single cells arranged in a layer on the inside of blood vessels and are the most abundant cells in lung tissue (40%) [11]. PMVECs are essential in maintaining the barrier function of the pulmonary microvasculature [12]. The pulmonary alveolar capillary barrier is formed jointly by PMVECs and alveolar epithelial cells (AECs) [13], the damage of either of which will have an impact on pulmonary functions. As the primary barrier, PMVECs are also adversely affected first. For example, reactive oxygen species (ROS) and oxidative stress are important mechanisms underlying the development of many lung diseases, while microvascular endothelial cells are critical target cells for ROS, which may have a higher sensitivity to ROS in the lung than in other tissues [14]. Normally, pulmonary capillaries are poorly permeable. However, where there is damage or apoptosis to PMVECs, cell dysfunction or defects will occur. As a result, there will be a change in the permeability of pulmonary capillaries [15]. Increased pulmonary microvascular permeability is a common characteristic of many pulmonary diseases [16–18].

Apoptosis and injuries of PMVECs have been reported to play an essential role in the development of numerous lung diseases. Pulmonary edema is a hallmark pathological feature of ALI [19], and pulmonary microvascular permeability is an important cause of noncardiogenic pulmonary edema [20]. Evidence has shown that diffuse PMVEC injury causes destruction of the pulmonary microvascular endothelial barrier, and enhanced permeability is responsible for the interstitial edema associated with ALI [21]. In a study on sepsis, lipopolysaccharide (LPS) accelerated the rate of apoptotic cell death in PMVECs via activation of yes-associated protein, leading to microvascular hyperpermeability and ALI under sepsis [22]. Microvascular injuries have also been recognized as an important event in the evolution of PF [23]. A few subsequent empirical studies of PF have confirmed this conclusion through the specific marker detection of PMVEC lesions [24], cell morphology and activity [25]. In the natural course of early COPD, the pulmonary microvascular blood flow is overall reduced in any of the subjects accompanied by apoptosis of PMVECs [26]. A previous study demonstratedthat cigarette smoke extract induces apoptosis of PMVECs by decreasing mitochondrial respiration in cell models, which in turn induces the occurrence of COPD [27]. This demonstrates that changes in pulmonary microvascular permeability caused by pulmonary microvascular damage are a common feature of many pulmonary diseases and may be a very important inducing factor.

PH is a progressive disease of the pulmonary microvascular system, with the fundamental pathological features of progressive loss of pulmonary microvessels and remodeling of pulmonary microvessels [28, 29] as well as abnormal vasoconstriction [30]. Remodeling of distal pulmonary vasculature refers to the structural changes in the blood vessels of the pulmonary artery branch region, including the narrowing of pulmonary arterioles, thickening of the muscular layer, and proliferation of endothelial cells. The thickening of pulmonary arterioles and inappropriate pulmonary vasoconstriction restrict the normal flow of pulmonary blood, resulting in increased pulmonary arterial pressure and further exacerbating the pathological changes in pulmonary vasculature. The increase of pulmonary vascular resistance caused by distal pulmonary vascular remodeling is a key cause of increase of pulmonary artery pressure [31].

PMVEC dysfunction is believed to be at the root of PH [32]. During pregnancy, changes in certain proinflammatory cytokines or vascular signals in pregnant women may promote the development of alveolar septal fibrosis and impair the development of pulmonary microvasculature, thus leading to neonatal PH [33]. And models induced by the endothelial toxin monocrotaline (MCT) and hypoxia are commonly used in experiments established in animal models of PH, both of which have been shown to trigger initial apoptosis and damage to endothelial cells [34–36]. When the PMVECs show abnormal endothelial functions due to other factors, it may lead to pulmonary vasoconstriction and remodeling of the distal pulmonary vasculature through the following pathological alterations:

(1) Pulmonary microvascular permeability: Under pathological conditions such as a high glucose state, inflammatory infiltration, hypoxia or hyperoxia, PMVECs are subjected to damage, leading to apoptosis. This results in the release of injury markers such as CD31, as well as pro-inflammatory cytokines like tumor necrosis factor-alpha (TNF-α) and interleukin-6 (IL-6). Additionally, the expression of the cell adhesion protein VE-cadherin is decreased, thereby compromising the structural integrity and adhesive capacity of PMVECs; this phenomenon leads to a bare capillary basement membrane [37–41] and thus increases pulmonary microvascular permeability. In addition, inflammatory cells and platelets synthesize and release large amounts of vascular endothelial growth factor (VEGF) under inflammatory conditions [42], which can significantly enhance vessel permeability [43]. When the pulmonary microvascular permeability increases, proteins, inflammatory cells, and inflammatory factors can leak into the surrounding tissues [44–46]. Several inflammatory factors, including PDGF, TNF-α, IL-6, IL-8, and TGF-β, among others, can leak and stimulate excessive proliferation of smooth muscle cells. PDGF is a potent cell proliferation factor that can increase smooth muscle cells by a factor of 2.3 in 48 h by upregulating transient receptor potential channel protein 6 (TRPC6) [47]. In animal models of PH, it has been found that the expression level of platelet-derived growth factor receptor-beta (PDGFR-β) significantly increases and is an important factor in promoting smooth muscle cell proliferation through pulmonary microvascular leakage [48, 49].

(2) Imbalance in the release level of the regulator of vasomotor activity: Under normal conditions, PMVECs synthesize and secrete various vascular regulatory factors, playing a crucial role in the regulation of vascular dilation and constriction.

NO is a biologically active gas molecule synthesized by nitric oxide synthase (NOS) in endothelial cells, neurons, and other cells. It possesses diverse biological functions, including vasodilation, inhibition of platelet aggregation, and anti-inflammatory effects. NO can promote vasodilation by relaxing smooth muscle cells, thereby increasing blood flow and improving vascular function. In PH disease, it has been found that the production of endogenous NO in the lung is reduced [50, 51].The synthesis of NO is influenced by many factors. The stable expression of the mechanosensitive cation channel Piezo2 is essential for maintaining intracellular calcium influx and NO synthesis in PMVECs. When there is an imbalance between the TGF-β and bone morphogenetic proteins (BMP) signaling pathways, the expression of Piezo2 is reduced, leading to decreased phosphorylation of NOS and reduced NO synthesis. Consequently, this may weaken the inhibitory effect of cGMP-mediated smooth muscle cell proliferation, resulting in increased pulmonary muscularization [52].

Cav-1 is mainly expressed in PMVECs and plays an important role in maintaining pulmonary microvascular permeability and development [53, 54]; it can directly bind to the endothelial nitric oxide synthase (eNOS) and regulate its activity and stability by affecting the phosphorylation status of eNOS, further modulating its function [55]. The abnormal expression and dysfunction of Cav-1 protein is associated with PH [56, 57]. In the study of PMVECs, it was found that under the influence of inflammatory mediators such as IL-6, PMVECs were damaged, resulting in the loss of the expression of Cav-1 protein and the uncoupling of eNOS, failure to synthesize NO normally, and excessive peroxynitrite, which led to the loss of BMP receptor-2 (BMPR2), and activated TGF-β/pSMAD2/3 signaling pathway, thus forming pulmonary vascular remodeling [58]. BMPR2 is an anti-apoptotic factor of endothelial cells, and its mutation and loss is an important genetic basis for PH [59, 60].

Hyperconstriction of pulmonary blood vessels is another important pathological feature of PH. Current research indicates that ET the strongest arteriovenous vasoconstrictor peptide [61], which is produced by the breakdown of big endothelin by endothelin converting enzyme on endothelial cells [62]. ET-1, one of the three isomers of ET, is mainly synthesized by endothelial cells and is a vasoactive factor regulating pulmonary vascular tension [63]. Its combination with ET-1 receptor type A (ETA) in pulmonary blood vessels can lead to pulmonary vascular contraction, smooth muscle cell proliferation and pulmonary fibrosis [64]. Under physiological conditions, ET-1 levels were maintained at low levels due to NO inhibit the expression of the ET-1 and the scavenging effect of the ET-1 receptor type B (ETB) in the lungs [65, 66]. Elevated ET-1 levels have been observed in both clinical PH patients and experimental PH animal models in lung tissue and systemic plasma [67, 68]. Moreover, in most PH patients, the clearance effect of ETB did not change, so excessive synthesis is the main reason for the increase of ET-1 [69].The main source of increased ET-1 synthesis in PH is PMVECs [70]. In in vitro experiments, TGF-β and BMP9 can promote the synthesis of ET-1 by inducing the phosphorylation of Smad1/5 in human PMVECs, among which BMP9 is more effective and rapid, and can induce the phosphorylation of Smad2 at the same time [71, 72]. BMPR2 loss or knockdown can promotes the signaling effects of BMP9 and TGF-β in PH [73].

## The interaction between AECs and PMVECs

AECs, comprised of alveolar type I (ATI) and type II epithelial (ATII) cells, play an important role in maintaining lung integrity and homeostasis. These cells begin to appear during the tubule-forming stage of lung development; among them, ATI cells are flat and cover more than 95% of the gas exchange surface. The ATI cells not only facilitate the ejection of sodium and water from the alveoli but are also involved in removing the amino acids from the airway [74]. These cells cannot proliferate; they are replaced by ATII cells that transdifferentiate into ATI cells when they are injured [75]. ATII cells are also regarded as progenitors of the alveolus and play an important role in the lungs innate immune response [76]. ATII cells can secrete surfactant, which reduce alveolar surface tension and prevent alveolar collapse [77]. In the mature lung, the alveolar walls are covered by squamous ATI cells and round secretory ATII cells. Although ATII cells occupy only a small surface area, they far outnumber ATI cells [78].

The alveolar epithelium and its adjacent pulmonary microvascular endothelium, as well as the extracellular matrix between them, together constitute the alveolar-capillary barrier and maintain the normal fluid exchange and gas exchange of the alveoli. The relationship between alveolar epithelium and pulmonary microvascular endothelium is very close, and the two influence and maintain each other. Because of the complexity of the pulmonary alveolar system, we know little about the interactions between AECs and PMVECs, but many in vitro culture experiments on ATII cells and PMVECs have shown epithelial-endothelial interactions.

Stimulated by endotoxins, pro-inflammatory cytokines, and plasma of sepsis patients, the protein permeability of human PMVECs increases. However, ATII cell line A549 can counteract this damage by releasing soluble factors suspected to be lipids, thereby reducing the permeability of human PMVECs stimulated by endotoxins by 39 ± 4% to 100 ± 3% [79]. This repairing effect of AECs on PMVECs was further confirmed in the study. Studies have shown that under normal physiological conditions, A549 cells and ATI-like cells release lipid mediators S1P and maintain the barrier function of human PMVECs by binding to S1P1 receptors, while under the stimulation of endogenous toxins, A549 cells and ATI-like cells increase the release of lipid mediators S1P and PGE2. Binding to the S1P1 receptors and EP4 receptors mediates the effect of enhancing the barrier function of human PMVECs [80]. In addition, an interesting phenomenon was found in this study: dexamethasone could block the release of PGE2 in A549 under normal conditions but could not block the release of PGE2 in ATI-like cells, which may indicate that there are other pathways in ATI cells to synthesize PGE2. Alveolar epithelial cells are involved in repairing the barrier function of PMVECs by releasing S1P. In previous studies, PMVECs have been shown to be able to release S1P in the case of lung injury. By binding the S1P receptor S1PR2 on the alveoli and initiating nuclear translocation of the transformation regulator yes-associated protein (YAP), the transformation of ATII cells into ATI cells is promoted [81]. Therefore, whether ATII cells will promote their own transformation through SIP to complete repair is expected to be further proved by relevant studies. Since 2019, the severe acute respiratory syndrome coronavirus 2 (SARS-CoV-2) has been spreading globally. ATII cells represent the primary cellular targets of SARS-CoV-2 infection, while PMVECs also incur damage. Nonetheless, this impairment is not direct in nature; SARS-CoV-2 potentially instigates disruption within the mitochondria of ATII cells, subsequently precipitating an overabundance in the generation of ROS within these mitochondria. This sequence of events consequently stimulates the production of pro-inflammatory cytokines IL-1α, which are subsequently released into the intercellular space, thereby exerting an influence upon adjacent PMVECs. As a result, PMVECs undergo mitochondrial fragmentation, experience diminished Golgi apparatus integrity, and sustain compromised expression of the barrier-associated protein VE-cadherin [82]. In hypoxic conditions, AECII similarly exhibit deleterious effects on PMVECs. The study reveals that the ISM1 gene is upregulated in a low-oxygen environment. In comparison to the monoculture model of PMVECs, the co-culture model of PMVECs and AECII results in a further 37% increase in PMVEC permeability. This can be attributed to the overexpression of the hypoxia-inducible factor HIF-1α in AECII, which, by binding to regulatory elements, upregulates the expression of the permeability-associated gene ISM1, thus contributing to the enhanced PMVEC permeability [83].

**Fig. 1:**
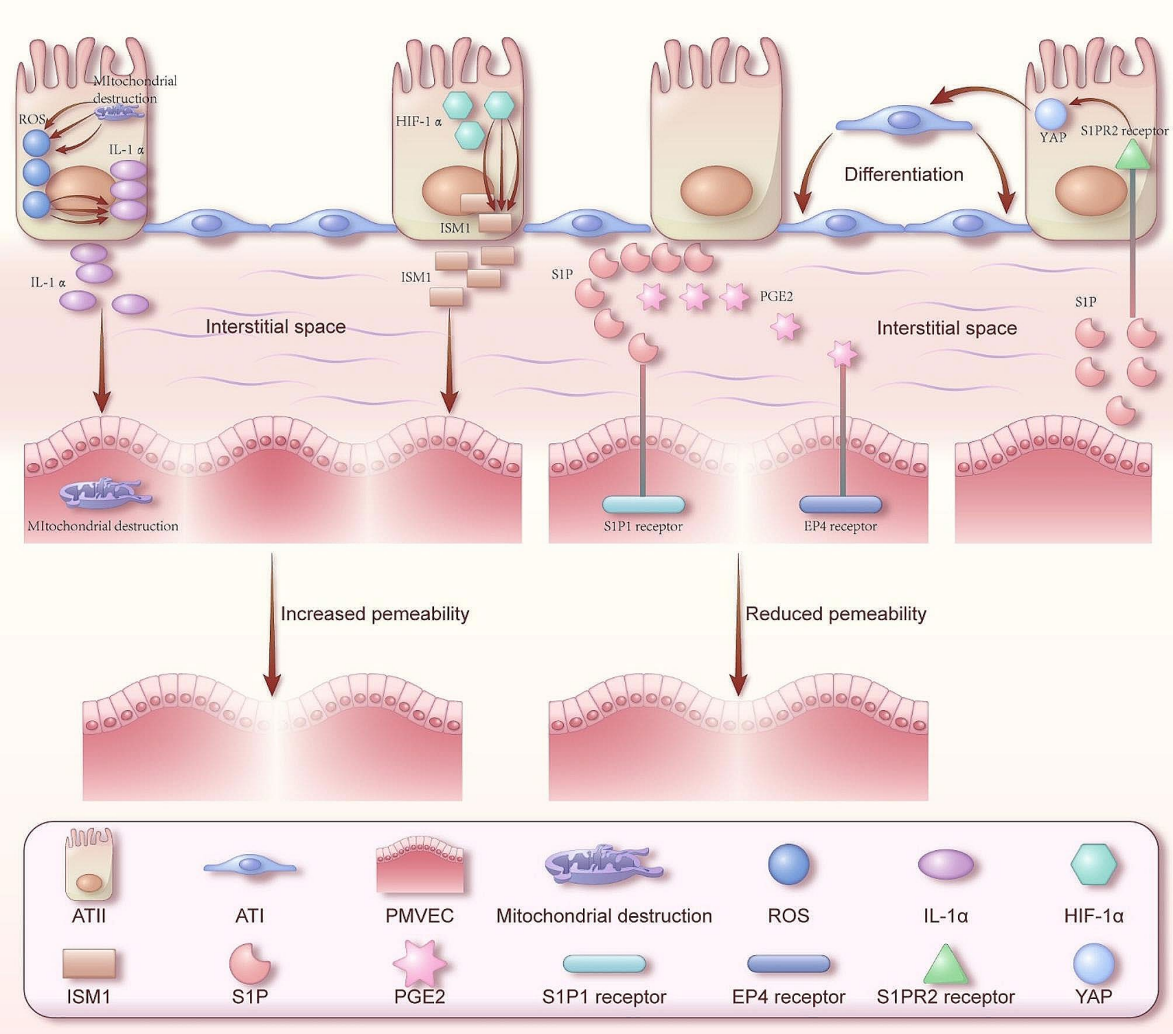
AECs and PMVECs interact with each other. Under conditions of injury, AECs have damaged organelles and dysfunction and regulate the permeability of pulmonary microvessels by secreting a number of mediators. The result of this adjustment will have different effects depending on the mediators. Similarly, PMVECs secrete lipids to affect alveolar repair in the state of injury. By Figdraw (www.figdraw.com)

In summary, there are complex interactions between AECs and PMVECs (Fig. 1). AECs and PMVECs maintain and interact with each other by releasing substances such as cytokines and lipids. When the body is in pathological surroundings, if the substances that regulate the secretion of ATII can favorably affect PMVECs by the paracrine effect of AECII, the damage to PMVECs can be reduced and the endothelial cell function of PMVECs can be repaired. The PMVECs that function normally can in turn promote the transformation of ATII to ATI and then repair injury of the lungs. Therefore, intervening in the abovementioned secretion will be an effective practice for the treatment of certain lung diseases including PH.

## Some problems in the current treatment of PH

The drug treatment system for PH is very complex, with many side effects and lack of sufficient stability. Patients usually receive more than one drug to treat PH. By directly regulating endothelial cells or acting on pulmonary artery smooth muscle cells (PASMCs), the drugs regulate blood vessels, either inhibiting vasoconstriction or promoting vasodilation, but they are unable to reverse vascular remodeling or restore the function of endothelial cells and AECs aiming at improving right ventricular function, that is, treating PH by addressing the root cause. Some medications require chronic use, resulting in other issues, such as decreased patient compliance, unknown long-term efficacy, and impaired organ function. In addition, PH primarily affects the arteries in the lungs, which is characterized by diffusion impairment, making it difficult for the drug to reach the intended area [84].

The currently available surgical options for the treatment of PH mainly include balloon pulmonary angioplasty (BPA), pulmonary artery thromboendarterectomy (PTE), and lung transplantation. These options have significantly improved patients’ symptoms and quality of life. However, some challenges remain with these options. PTE is currently the most effective and the preferred option for the treatment of CTEPH. Some patients with CTEPH can be completely cured with this approach. However, it is highly demanding for the surgeon’s techniques and experience, and 40% of the patients would not be indicated for this procedure as the position of the thrombus is difficult to reach [85]. BPA can significantly improve the patient’s symptoms and hemodynamic indicators, yet is associated with the complications primarily including pulmonary vascular injury and reperfusion pulmonary edema [86]. The 5-year survival rate following lung transplantation for PH is 45–50%; however, it is limited by the small number of donors and a high risk.

## Possible role of stem cell therapy in the repair of PH

In recent years, new research has been conducted on the role of stem cells in the treatment of lung diseases. Currently, more than 50 clinical trials involving stem cell therapies for pulmonary diseases have been registered on ClinicalTrials.gov. Over the last years, much progress has been made in the research field of stem cell therapy in PH. Currently, there are three major groups of commonly studied stem cells: embryonic stem cells (ESCs), induced pluripotent stem cells (iPSCs) and mesenchymal stem cells (MSCs).

### Embryonic stem cells

ESCs originate from the germinal vesicle, the earliest phase of embryo development. Specifically, ESCs are undifferentiated cells in the blastemal in early embryonic development (5th to 7th days after fertilization) and are characterized by infinite proliferation, self-renewal and a multidirectional differentiation capacity [87].

As mentioned above, ATII cells can promote the repair of the barrier function of pulmonary microvascular endothelial cells and reduce the permeability of pulmonary microvascular endothelial cells through paracrine effect, so transplantation of ATII that can secrete specific substances can be a therapeutic approach. Therefore, cell purity is the key problem to be solved first. Regardless of whether the tissue is cultured in primary or differentiated from ESCs, it is difficult to obtain sufficient purity and quantity of ATII cells. The production of ATII cells isolated from primary lung tissues is often low, prone to phenotypic loss, and accompanied by a loss of proliferative capacity [88, 89]. The use of ESC differentiation to obtain ATII cells has been studied for a long time.

In 2002, Ali et al. cultured embryoid bodies (EBs) derived from the differentiation of mouse ESCs with serum-free small airway growth medium (SAGM). On the 3rd and 14th days of culture, surfactant-protein-C (SPC) mRNA, a specific marker of ATII cells, was detected in cells. The purity of cells obtained in the experiment was quite low, and it relied heavily on the formation of EBs in the early stage. Moreover, a long period of time is required for differentiation. Nevertheless, this study revealed the possibility of ESCs differentiating into lung tissue [90]. In the follow-up study, the team tried to raise the output and realize more accurate differentiation status by recombining the effective components in SAGM. After removal of retinoic acid and triiodothyronine, the expression level was increased threefold. However, there was a large deviation in the repeatability of the data [91]. Although the addition of activin A could increase the output, the positive rate was quite low, as shown by the flow results, and the complete culture process took 35 days [92]. In 2007, our research team used a disaggregated seeding method to shorten the culture time required for detection of SPC mRNA transcripts without EB formation and pioneered a genetic screening directed differentiation procedure for human embryonic stem cells (hESCs), screening ATII cells with 99.6% purity and validating their cellular functions, cell biological features and morphological characteristics, as well as the expression of surfactant-protein-A (SPA), surfactant-protein-B (SPB) and SPC, which was detected by immunofluorescence staining, and the synthesis and secretion of the complement proteins C3 and C5 were detected on the 15th day of cell differentiation, which provided a reliable source of support for the late application of ATII cells [93]; cells obtained in this way show intervention and protection in ALI while recovering weight and arterial blood oxygen saturation, along with improved survival [94]. The differentiation of ESCs in vivo or in vitro is also regulated by the microenvironment [95, 96]. SPC protein expression was detected after 5 days of coculture with mouse lung embryonic mesenchymal cells, but this positive reaction did not occur when cocultured with mouse intestinal embryonic mesenchymal cells, indicating that the lung microenvironment may be more suitable for promoting the differentiation of embryonic stem cells into ATII cells [97].

ESCs can also differentiate into vascular endothelial cells. FOXF1 is a transcription factor that plays a pivotal role during embryonic development [98]. FOXF1 and its transcriptional target protein c-KIT are expressed in endothelial progenitor cell populations within the human pulmonary microvasculature [99]. In a study published in June this year, researchers used electroporation to introduce the FOXF1 and c-KIT genes into a mouse ESC line. Subsequently, they directed the differentiation of these stem cells into endothelial cells (CD31 + CD45-). Notably, these c-KIT + FOXF1 + EPCs, when transplanted into a mouse model, integrated into the alveolar microvasculature, expressing the alveolar capillary endothelial specific markers CAP1 and CAP2. The study also revealed that, in the lung tissue of mice transplanted with c-KIT + FOXF1 + EPCs, donor cells in the distal lung regions co-expressed early lung epithelial cell markers such as Nkx2.1(TTF1), SRY-box transcription factor 2 (SOX2), and SRY-box transcription factor 9 (SOX9). This suggests that the donor cells contain not only endothelial progenitors but also early lung progenitors, holding the potential to generate multiple types of lung cells in the recipient lungs. In addition, it is important that c-KIT + FOXF1 + EPC obtained through directed induction differentiation can effectively implant pulmonary microvascular in mice with hyperoxy-induced injury, successfully increasing the number of endothelial cells and restoring the density of pulmonary microvessels [100]. However, the differentiation method used in this research achieved only a 4% yield of endothelial cells.

The differentiation of ESCs into endothelial cells is regulated by transcription factors such as HIF. As a critical transcriptional regulator of lung tissue sensing oxygen changes in a hypoxic environment, HIF can regulate the expression of a range of hypoxia-related target genes and affect the process of pulmonary vascular remodeling. IL-33, a key factor in multiple types of pulmonary inflammation and vascular remodeling, promotes pulmonary vascular remodeling by increasing the expression of HIF-1α in PH [101]. Interestingly, some studies have demonstrated that HIF-1α promotes the differentiation of ESCs into functional vascular endothelial cells by initiating the transcription of ets variant 2 (ETV2) and simultaneously activating the NOTCH1 signaling pathway [102].

Therefore, the differentiation of ESCs into ATII cells or PMVEC by directional induction comes with significant research prospects. In summary, the following two pathways may be available for treating PH with ESCs: ① ESCs can directionally differentiate into normal ATII cells or PMVEC and restore the signal transduction between endothelium and epithelium through their interaction, thus restoring normal barrier function and alveolar epithelial function; ② ESC-derived ATII cells or PMVECs can be modified to perform specific functions against different pathogenic factors using gene-modification techniques, such as restoring the sensitivity of endothelial progenitor cells to hyperoxic environment and stimulating neovascularization and alveolization under hyperoxic environment. In addition to their therapeutic potential, ESCs can also be used in combination with gene editing techniques to create different models of PH, including the hereditary form of the condition. This approach allows researchers to investigate the roles of various genes, which, in turn, provides significant assistance in the study of targeted treatments for PH.

ESCs have the unique properties of unlimited self-renewal and pluripotency, this ESCs’ primary advantage is also its primary disadvantage. As mentioned above, the differentiation of ESCs is affected by the microenvironment. Teratomas can form when ESCs are in an ectopic microenvironment [103]. Overall, tumorigenicity is a potential hurdle that could halt ESC research. ESC-expressed Ras is an identified oncogene also required for endothelial differentiation of mouse ESCs [104]. MicroRNA-mediated regulation of Sox2 is an embryonic stem cell-specific transcription factor that controls self-renewal, pluripotency, and proliferation [105] and has been shown to function as an oncogene [106]. These problems must be ruled out, and the development of tumors must be avoided by preparing highly pure, directionally differentiated cells. In addition, potential immunological rejection and chromosomal variation are critical limitations [107–109]. Although our research team has made cells resistant to CD8 + T-cell-mediated destruction by targeting blockade of human leukocyte antigen (HLA) light chain-β2 microglobulin expression without affecting the self-renewal capacity, genomic stability or pluripotency of hESCs, this has only minimized hESC immunogenicity. The production of completely immunogenic hESCs continues to be a major challenge. Furthermore, due to the particularity in defining and sourcing hESCs, their clinical application is surrounded by ethical controversy.

Some scholars argue that an embryo should be considered a human person from the moment of conception, and thus acquiring hESCs through embryo destruction is tantamount to murder. Therefore, regardless of the ultimate goal of hESC research, such disregard for life is unacceptable. On the other hand, supporters of hESC research believe that the definition of humanity should include social attributes. During the first 14 days of development, embryos have no nerves or brain, and at this stage, embryos are devoid of consciousness and the ability to engage in social relationships. Therefore, extracting ESCs during this period, before the embryo begins to develop its bodily plan, should be permitted. Embryos at this stage should not be defined as complete individuals but rather as “a potential human being,” symbolizing a form of “special respect.” However, this notion continues to face widespread opposition [110].

To balance technology and ethics and promote the development of hESC technology, many countries around the world have enacted their own regulations on hESC research, but the stringency varies greatly between different countries. Germany has always maintained a very conservative attitude towards the application and research of hESCs. It wasn’t until December 2002 that the German government first approved the entry of hESC lines into Germany. Before this, Germany had explicitly prohibited any hESC research by enacting the Embryo Protection Act [111]. Influenced by political factors, the United States has been cautious in the legal regulations of hESC research. The U.S. has long restricted funding for hESC research and prohibited the production of any hESC strains that require embryo destruction. Research using hESC strains is limited to those created before August 9, 2001, and regulation of hESC research projects funded by private or non-federal funds is relatively lenient [112]. The United Kingdom is one of the earliest countries in the world to legislate and regulate hESC research. After a prolonged period of ethical debate, the government has formulated relatively enlightened and comprehensive regulatory policies for hESC research. In 1990, the UK enacted the Human Fertilisation and Embryology Act, which, by reference to the “14-day” principle proposed by the US Department of Health, Education, and Welfare Ethics Advisory Board in 1979, permits the in vitro creation, preservation, and utilization of hESCs for research purposes. However, the ESCs used in research cannot develop in vitro for more than 14 days, and all hESC research conducted in the UK is only legal with approval from the Human Fertilisation and Embryology Authority (HFEA) [113]. Unlike Western religious beliefs, in China, influenced by millennia of doctrine, most people do not consider embryos sacred from the moment of conception. However, this does not mean that the Chinese do not respect embryos; rather, they believe that embryos have a special moral status. When they can be used to treat diseases, the Chinese can tolerate the destruction and use of human embryos. Therefore, research on hESCs is supported in China, and in recent years, this research direction has been designated as a key focus of national research and development plans. In terms of regulation, China primarily relies on the Measures for the Ethical Review of Biomedical Research Involving Human Subjects and the Ethical Guidelines for Human Embryonic Stem Cell Research to impose ethical restrictions on research involving hESCs. However, there is no comprehensive legal framework to regulate the research on hESCs or to penalize research activities that violate regulations. Since the “gene-edited babies” incident in 2018, China has been continuously revising its laws to improve its regulatory methods, but there is still a long way to go [114].

Currently, one of the unresolved issues may be how to reasonably obtain hESCs, ensuring that their sources comply with medical ethics and reach a unified consensus on religious beliefs. In addition, comprehensive regulations are essential to provide normative guidance for hESC research.

### Induced pluripotent stem cells

First reported by Kyoto University in Japan in 2006, iPSCs are now widely used in the study of respiratory diseases. Transplantation of ATII cells formed by the differentiation of human iPSCs into a rat model of pulmonary fibrosis was shown to effectively inhibit the increase in α smooth muscle actin (α-SMA) and transforming growth factor-β (TGF-β) and reduce collagen content, thereby reducing pulmonary fibrosis and improving the alveolar structure [115]. In addition, a number of studies have confirmed the ability of iPSCs to battle ALI. In an experiment by Dongqi Xing et al., intravenous infusion of iPSC-derived pulmonary vascular endothelial cells expressing CXCR1/2 or CCR2/5 from rats significantly inhibited ALI induced by LPS [116]. Vincent Yi-Fong Su et al. showed that iPSCs reduced adhesion kinase activity via tissue inhibitors of metalloproteinase 1 (TIMP-1) and focal adhesion kinase (FAK)/Snail pathways and reduced LPS-induced histological signs, protein leakage and proinflammatory cytokine expression in ALI, as well as improved endothelial cell permeability [117].

The technique of creating iPSCs has good therapeutic potential, as it may enable the replacement of malfunctioning organs with newly differentiated cells. In addition to already being used to derive respiratory epithelial cells, vascular endothelial cells, and vascular smooth muscle cells [118, 119], iPSCs have also been applied in the study of lung development and vascular modeling [120].

In recent years, the role of IPS in PH has also begun to emerge in the field of pulmonary disease research. In an MCT-induced PH rat model, iPSCs and iPSC-conditioned medium could improve hemodynamics and right ventricular hypertrophy and significantly reduce the expression of the inflammatory markers CD68, MHC-II, IL-1β, IL-6, IL-12α, IL-12β, IL-23 and interferon γ [121].

In terms of differentiation, iPSCs also show potential for the treatment of PH. IPSC-derived ECs (iPSC-ECs) have many similarities with natural pulmonary arterial endothelial cells and can be used as substitutes for drug screening in PH. In animal experiments, IPSC-differentiated ECs were injected into the pulmonary vascular lumen of rats, restoring 85–95% of pulmonary microvascular barrier function and regenerating functional blood vessels [122]. ATII cells derived from iPSCs, which were obtained by Bernard Thébaud’s research team, with a 96% expression rate of SPB positivity at day 25, were screened and transplanted via the airway to prevent lung injury produced by high oxygen concentrations in mice [123]. The differentiation of iPSCs into ATII cells is not very efficient. IPSC-ATII overexpressing miR-22 was found to have increased SPC-positive cell expression (49.12% vs. 37.21%) compared to fibroblast factor-2-induced ATII cells at day 5 of culture by Fan Tao’s research team for comparison but was still inferior in cell function to primary cultured ATII cells [124]. As with embryonic stem cells, tumorigenicity represents a major obstacle to the clinical application of iPSCs [125, 126]. Our research team used genetic modification to precisely express Oct4, Sox2, cMyc and Klf4, followed by transgenic removal of the exogenous reprogramming factors. The resulting iPSC-differentiated ATII cells are homologous and have the structure and biological function of normal ATII cells, making this technique effective in protecting and regenerating damaged AECs without being tumorigenic [127].

Therefore, it demonstrates that lung injury repair through differentiation into endothelial cells or ATII cells is also an effective approach for the therapeutic use of iPSCs to treat PH. Additionally, iPSCs can also contribute to the treatment of PH in other ways as follows: ① Disease modeling: Studies have shown that ECs differentiated from heritable pulmonary arterial hypertension (HPAH) patients-derived iPSCs have similar cell morphology to the donor’s pulmonary artery endothelial cells (PAECs) and also exhibit functional abnormalities such as decreased cell migration ability and diminished ability to generate blood vessels. Among 8 donors, 2 patients with HPAH-derived iPSC-ECs and PAECs showed a significant increase in blood vessels after treatment with the BMPR2 signaling activators FK506 and Elafin [128], indicating the feasibility of using iPSC-ECs as a substitute for PAECs in establishing PH models. ② Drug screening: Based on the above findings, the Caspase detection of cell apoptosis was utilized to screen drugs for PH iPSC-ECs. Among six compounds, some tyrosine kinase inhibitors (TKIs) were identified as superior to others, including imatinib, in improving PH vascular function [129]. ③ Individualized treatment: Using sequencing technology to analyze PAECs derived from idiopathic pulmonary arterial hypertension (IPAH) patients, as well as endothelial cells differentiated from iPSCs originating from fibroblasts, evaluating differences in gene expression and methylation changes, elucidating the relationship between variant genes and the pathobiology of PH. Subsequently, correcting genes through zinc finger nucleases technology and comparing changes in vascular function after restoring normal gene expression. This study may offer a direction for personalized therapy for PH [130].

Meanwhile, iPSCs are also applied in the study of protective modifiers for HPAH. Mutations in BMPR2 can be found in about 70% of patients with HPAH and around 20% of patients with IPAH [131]. It has been shown that genetic deletion of endothelial BMPR2 activates endothelial apoptosis and causes PH in endothelial cells [132]. For familial PH, however, only 20% of carriers with BMPR2 gene mutations exhibit clinical symptoms, indicating that the cellular function can be protected from BMPR2 mutations in the presence of certain protective modifiers. Moreover, the comparative analysis found that FPH-derived iPSC-ECs showed significant decreases in cell adhesion and survival compared to unaffected carrier-derived iPSC-ECs. At the same time, the RNA analysis revealed two compensatory signals, i.e., pP38 and BIRC3 [133]. Furthermore, as iPSCs share the same genetic background as the donors, which largely avoids the issue of immune rejection and boasts strong genetic operability, investigators can attempt to repair these mutations using CRISPR-Cas9 or other gene editing techniques, thereby developing potential gene therapies that provide significant clinical value in the treatment of patients with HPAH.

Previous results showed that iPSC-derived MSCs (iPSC-MSCs) exhibited stronger proliferation than MSCs [134], and iPSC-MSCs also appeared to have better therapeutic efficacy in animal experiments [135]. IPSCs can avoid many of the ethical dilemmas and the risk of immune rejection but might induce tumor generation. Beyond this, obtaining high-quality patient-specific iPSCs requires considerable time and expense, and the technical aspect is also a major challenge. Previous studies have reported that it takes at least 3 weeks from the start of induction to the establishment of stable iPSCs, and the induction efficiency is less than 1/5000 [136–138].

### Mesenchymal stem cells

MSCs are derived from the mesoderm and can undergo self-renewal and exhibit multipotency, along with multidirectional differentiation potential. In 2006, the International Society for Cellular Therapy (ISCT) listed three minimum standards for MSCs as follows: (1) show adherence and growth; (2) must express specific surface markers, including CD73, CD90 and CD105, without the expression of hematopoietic antigens, such as CD14, CD34, CD45 or CD11b and Human Leukocyte Antigen-antigen D Related (HLA-DR); and (3) have the capacity to differentiate into adipocytes, osteoblasts and chondrocytes [139]. MSCs are present in all tissues of mesenchymal origin and exist primarily in bone marrow. Currently, the categories of MSCs that are widely used in research primarily include bone marrow mesenchymal stem cells (BM-MSCs), adipose-derived mesenchymal stem cells (AD-MSCs), human umbilical cord mesenchymal stem cells (hUC-MSCs), human placenta mesenchymal stem cells (hPMSCs), dental pulp mesenchyme stem cells (DPSCs) and so on [140]. The above MSCs exhibit self-renewal, multilineage differentiation potential, low immunogenicity [141], and immunomodulatory properties. These features are regulated by a cascade of transcription factors [142–145]. In addition, growth factors and cytokines as well as RNA take part in the regulation of MSCs [146–148]. MSCs mainly exert their therapeutic effects through the secretion of paracrine and autocrine factors for tissue repair, tissue regeneration, anti-inflammation, inflammation reduction, and anti-apoptosis [149, 150], among others.

MSCs are an effective method for treating lung diseases, including ALI, COPD and PF. In an inflammatory environment, MSCs can secrete different factors, such as interleukin-2, which enhances regulatory T cells [151] (or through paracrine signaling by releasing anti-inflammatory mediators such as IL-10, PGE2, and ang-1) and repairs ALI, and MSCs can relieve inflammation, reduce the number of goblet cells, regenerate lung tissue, ameliorate the loss of alveolar septa, and reduce the levels of NF-κB and p65 in the lung tissue of rats with COPD [152]. In addition, MSCs can inhibit the synthesis of TGF-β1 in AECs in pulmonary fibrosis by releasing Stanniocalcin-1 (STC1), reducing the immune function of macrophages, and repairing the incorrect epithelial-mesenchymal transition relationship [153].

Similarly, many studies have demonstrated the therapeutic potential of MSCs for PH. MSCs are widely used in preclinical studies for the treatment of PH. In both rat and mouse disease models, MSCs have been observed to alleviate and improve PH symptoms [154]. Although the mechanism is unclear, there are three aspects that can be further studied.

First, MSCs have immunoregulatory effects on PH. In the PH model induced by MCT, multiple related signaling pathways of inflammation showed abnormalities, such as prostaglandins, endothelin and the NO pathway. MSCs can restore the signaling pathways and protect against vascular remodeling induced by PH and then effectively reduce the thickness of the vessel wall and perivascular fibrosis [155]. The excessive proliferation of PASMCs associated with inflammation is one of the mechanisms of PH. MSCs significantly reduce the production of tumor necrosis factor-α (TNF-α), calcineurin (CaN) activity and nuclear factor of activated T cells (NFAT)-c2 activity, thereby inhibiting the excessive proliferation of PASMCs [156].

Second, MSCs can repair PMVECs. In lung injury studies, MSC-derived microvesicles (MVs) in coculture systems were found to improve lung injury by promoting Ang1 mRNA and protein secretion and restoring impaired PMVEC permeability to glucose [157]. Likewise, the experimental results in ALI and ARDS studies show that MSCs support paracellular permeability recovery in PMVECs induced by LPS injury and reduce PMVEC apoptosis [158]. In PMVECs, the STAT3 signaling pathway helps upregulate the expression of certain induced proinflammatory cytokines associated with ALI and ARDS [159]. UC-MSC-derived exosomes inhibit STAT3 signal transduction and upregulate miR-204 expression in PMVECs under anaerobic PH conditions; miR-204 is a critical microRNA with a reduced expression level in human PH [160]. In addition, UC-MSC-derived exosomes can significantly inhibit hypoxia-induced PMVEC apoptosis and PASMC proliferation, thereby suppressing pulmonary vascular remodeling and improving PH [161]. PMVECs support NO expression and can mediate autovasodilation. Endothelial nitric oxide synthase (eNOS) in PMVECs is essential to maintain normal endothelial cell function and homeostasis and plays a modulatory role in PH [162]. ADSCs can increase eNOS expression in PMVECs and enhance NO production in cocultures to improve PMVECs [163]. Binding of caveolin-1 (CaV1) with eNOS inactivates eNOS. Intravenous injection of MSCs carrying CaV1 mutants into the rat model of PH (1) significantly increases the NO level in the animal serum, (2) reduces the expression of α-SMA, and (3) reduces the thickness of pulmonary vascular tunica media [164]. Moreover, exhaustion of extracellular superoxide dismutase (ecSOD) in pulmonary MSCs was shown to accelerate pulmonary microvascular remodeling in PH, which is regulated by the typical Wnt/β-catenin pathway [165].

The third point is the effect on AECs. In the rat model of ALI, MSCs and ATII showed the same therapeutic effect [166]. Under air-liquid culture conditions, MSCs showed similar morphology and function to AECs, which proved that MSCs have the potential to differentiate in this direction [167]. This differentiation process is regulated by signaling pathways. Through a study on the treatment of ARDS, researchers found that when the Wnt/β-catenin, Wnt/JNK, or Wnt/PKC signaling pathways are activated, this differentiation ability will be promoted [168, 169]. In contrast, once the Wnt5a pathway is inhibited, such a differentiation process will be blocked [170]. In addition, the action of positive regulatory genes not only promotes the differentiation of MSCs into AECs but also increases the retention of MSCs in the lungs. In addition to differentiation, MSCs can also reduce the expression of HIF-1 and ROS proteins, inhibit the apoptosis of AECs and play a protective role by interfering with hypoxia stress signals [171].

Extracellular vesicles (EVs) may play a crucial role in some of the therapeutic effects of the above MSCs. EVs are nanosized particles released by cells that are present in most tissues and biological fluids, such as serum, cerebrospinal fluid, urine and cerebrospinal fluid [172], and can also be obtained in cell culture medium. EVs were initially considered as garbage bags of cellular waste, which were used to dispose and collect cellular waste. Later, EVs were proved to stably carry many important signaling molecules, which are involved in important physiological processes such as angiogenesis, cell migration and cell growth [173–175]. Importantly, EVs also function as messengers between cells, such as drug carriers that carry important cellular signals. Extracellular vesicles mainly consist of four subgroups: exosomes (30–150 nm), MVs (50–1000 nm), apoptotic bodies (500–2000 nm) and oncosomes (1–10 μm) [176]. At present, the research focus is the exosome subgroup. They can transfer substances including DNA, RNA or proteins between cells and thereby influence the function of the recipient cells. For the above therapeutic effects of MSCs, paracrine effects contribute significantly, and these cells are able to express, synthesize, and secrete various bioactive molecules such as various growth factors, cytokines, regulators, and signal peptides, as well as regulate active factors involved in metabolism, immunity, cell differentiation, proliferation, migration, nutrition, and apoptosis, through which a balance has been achieved for the homeostasis of the body, thus providing a suitable environment for stem cell immune regulation and anti-apoptosis [177–179]. Afterward, we organized the results on MSC-derived EVs in the treatment of PH into Table 1.

**Table 1:** Studies of stem cell-derived EVs in PH

Source	Model	Dose, Time	Route	Result	Reference	
MSCs	Rat, Su/Hx	100 µg/kg, once every 5 days	i.v.	RVSP ↓; RV/LV + S ↓	[180]	
BM-MSCs	Mouse, MCT	25 µg/day, 3 days	i.v.	RV/LV + S ↓; WT/D ↓	[178]	
UC-MSCs, BM-MSCs	Mouse, HYRX		i.v.	RVSP ↓; suppress inflammation by immunomodulation of macrophage phenotype	[181]	
BM-MSCs	Rat, nitrofen	1 × 10^10^/ml	i.v.	attenuate pathological ECM remodeling; MMP-9 ↓	[182]	
UC-MSCs, BM-MSCs	Rat, HYRX	2.0 × 10^8^/g 12 × 10^8^/g 60 × 10^8^/g	i.v.	RVSP ↓; RV/LV + S ↓; medial wall thickness of pulmonary vessels ↓	[183]	
UC-MSCs	Mouse, HYRX	0.5 × 10^6 ^cell or 1.0 × 10^6 ^cell	i.v.	vascular muscularization and collagen deposition ↓; RV/LV + S ↓	[184]	
UC-MSCs	Rat, MCT or Hx	25 µg/day, 3 days	i.v.	RVSP ↓; RV/LV + S ↓; attenuate PH pulmonary vascular remodel; wnt5a, RhoA and GSK3β/β-catenin ↑	[189]	
UC-MSCs	Rat, MCT	25 µg/day, 3 days	i.v.	RVSP ↓; RV/LV + S ↓; attenuate PH pulmonary vascular remodel; medial wall thickness of pulmonary vessels ↓; α-SMA ↓;VE-cadherin ↑	[185]	
BM-MSCs	Rat, MCT	30 ug/100 ul/alter- nate days, 2 weeks	i.v.	RVSP ↓; RV/LV + S ↓; attenuate PH pulmonary vascular remodel	[186]	
BM-MSCs	Rat, MCT	30 ug/500 ul/ every two days, 5 weeks	i.v.	medial wall thickness of pulmonary vessels ↓; RV/LV + S ↓	[187]	
MSCs	Rat, Su/Hx	20 ug/kg/week, 5 weeks	i.v.	RVSP ↓; RV/LV + S ↓	[188]	
MSCs	Mouse, MCT	25 ug/100 ul/week, 4 weeks	i.v.	attenuate PH pulmonary vascular remodel	[154]	

MSCs are adult stem cells with poor specificity, and their ability to differentiate into AECs or PMVECs needs further confirmation and exploration. Fundamentally treating PH by intervening in AECs or PMVECs through paracrine effects is an optimal strategy in the case of MSCs. Compared with ESCs and iPSCs, MSCs have weaker differentiation ability despite their advantages in terms of low immunogenicity. Exosomes are the main treatment tool for MSCs. In addition, for treating PH, the dose and frequency of MSCs remain to be clarified, without a completely reliable basis for clinical treatment, which has limited their application. A better strategy is to facilitate MSC exosome expression of certain specific proteins by gene editing to promote the treatment of PH.

The advantage of MSCs in clinical practice also lies in the homing phenomenon, i.e., targeting the damaged tissue [189]. However, this strategy is not a completely “sharpened sword”. This homing phenomenon is not accurate and efficient [190, 191]; instead, it is subject to many influencing factors [192], such as inflammation status [193], the number of ligands [194] and organismic age [195]. Studies have found that MSCs are virtually nontumorigenic and display a stable phenotype in long-term culture [196]; moreover, they pose a low risk of immune rejection [197, 198]. However, such immune escape is not absolute [199]. The poor specificity of MSCs and the side effects caused by the downregulation of immune function are also issues that need to be considered in clinical application. In addition, MSCs may accelerate the risk of pulmonary fibrosis [200, 201], which needs to be further confirmed. In a study performed at Hannover Medical School in Germany this year, a 3-year-old girl with severe PH due to hereditary hemorrhagic telangiectasia was injected by clinical researchers with 5 consecutive intravascular infusions of allogeneic MSCs over 6 months. The results showed significantly improved hemodynamic parameters and reduced markers of vascular injury and vascular inflammation, with no adverse side effects observed [202]. Although there are still many unknowns about the efficacy of mesenchymal stem cells in treating PH, this is undoubtedly exciting news.

## Conclusions

ESCs possess the broadest pluripotency, but their application is limited by ethical concerns and immunological rejection issues. IPSCs, generated from a patient’s own cells, have the potential for unlimited proliferation and hold possibilities for gene therapy to address HPAH. They also largely avoid ethical and immunological rejection issues. However, there are risks of genomic alterations during cell amplification in vitro, and the four reprogramming factors are associated with tumorigenicity. Therefore, complex techniques are required to fully address these safety concerns before using iPSCs. MSCs, due to their limited differentiation ability, are currently studied more for their paracrine effects. The specific mechanisms involved in treating PH are not fully understood, and treatment outcomes may vary among different patients. Moreover, it is necessary to further ascertain whether long-term application of MSCs is required and if any side effects may arise. Hence, better regulation and selection methods are needed. While there are numerous challenges to be resolved in using stem cell therapy for the treatment of PH, the therapeutic potential it holds is a compelling reason for further research and exploration.

Currently, more insight is needed into the underlying mechanisms in the study of PH. Early signs of PH are difficult to diagnose, and further research into stem cell-based PH therapy should focus on whether it can prevent or reverse the progression of persistent PH symptoms. PMVECs and AECs are two good entries, and more high-quality clinical research evidence is needed.

## Data Availability

All data is available on request.

## Abbreviations

PH: Pulmonary hypertension
mPAP: Mean pulmonary artery pressure
RHC: Right-sided heart catheterization
PAWP: Pulmonary arterial wedge pressure
PVR: Pulmonary vascular resistance
NO: Nitric oxide
ALI: Acute lung injury
COPD: Chronic obstructive pulmonary disease
ARDS: Acute respiratory distress syndrome
PF: Pulmonary fibrosis
PMVECs: Pulmonary microvascular endothelial cells
AECs: Alveolar epithelial cells
LPS: Lipopolysaccharide
BPD: Bronchopulmonary dysplasia
MCT: Monocrotaline
TRPC: Transient receptor potential cation channel
PKC: Protein kinase C
MAPK: Mitogen-activated protein kinase
cAMP: Cyclic adenosine monophosphate
VEGF: Vascular endothelial growth factors
VSMCs: vascular smooth muscle cells
PGI2: Prostaglandin-I-2
ET-1: Endothelin
5-HT: 5-hydroxytryptamine
S1P: Sphingosine-1-phosphate
S1PR2: Type II S1P receptor
BRCA1: Breast cancer 1
GREM1: Gremlin1
ang-1: Angiopoietin-1
BMPR-II: Type II bone morphogenetic protein receptor
HIF: Hypoxia-inducible factor
smad: Drosophila mothers against decapentaplegic protein
ATI: Alveolar type I
ATII: Alveolar type II
TD: Trans-differentiation
nCLD: Neonatal chronic lung disease
PDGF-Rα: Platelet-derived growth factor receptor α
PGE2: Prostaglandin E2
SARS-CoV-2: Severe acute respiratory syndrome corona virus 2
VE-cadherin: Vascular endothelial cadherin
ROS: Reactive oxygen species
IL: interleukin
IFN: Interferon
TER: Transendothelial electrical resistance
ISM1: Isthmin1
PDE-5: Phosphodiesterase-5
sGC: Soluble guanylate cyclase
ERAs: Endothelin receptor antagonists
PPHN: Persistent pulmonary hypertension in children
cGMP: Cyclic guanosine monophosphate
PASMCs: Pulmonary artery smooth muscle cells
PTE: Pulmonary thromboendarterectomy
ESCs: embryonic stem cells
iPSc: Induced pluripotent stem cells
MSCs: Mesenchymal stem cells
EB: Embryoid bodies
SAGM: Small airway growth medium
SPA: Surfactant-protein-A
SPB: Surfactant-protein-B
SPC: Surfactant-protein-C
C3: Complement 3
C5: Complement 5
ETV2: Ets variant 2
HLA: Human leukocyte antigen
α-SMA: α smooth muscle actin
TGF-β: Transforming growth factor-β
CXCR: Chemokine receptor
OCT4: Octamer transcription factor 4
SOX2: SRY-box containing gene 2
KLF4: Kruppel-like factor 4
ISCT: International Society for Cellular Therapy
HLA-DR: Human Leukocyte Antigen-antigen D Related
TIMP-1: Tissue inhibitors of metalloproteinase 1
FAK: Focal adhesion kinase
iPSC-ECs: IPSC-derived ECs
iPSC-MSCs: IPSC-derived MSCs
BM-MSCs: Bone marrow mesenchymal stem cells
AD-MSCs: Adipose-derived mesenchymal stem cells
hUC-MSCs: Human umbilical cord mesenchymal stem cells
hPMSCs: Human placenta mesenchymal stem cells
DPSCs: Dental pulp mesenchyme stem cells
STC1: Stanniocalcin-1
TNF-α: Tumor necrosis factor-α
CaN: Calcineurin
NFAT: Nuclear factor of activated T cells
MVs: Microvesicles
eNOS: Endothelial nitric oxide synthase
CaV1: Caveolin-1
ecSOD: Extracellular superoxide dismutase
Su/Hx: Sugen-5416/Hyposia
RVSP: Right Ventricular Systolic Pressure
RV: Right Ventricle
LV + S: Left Ventricle + Septum
MCT: Monocrotaline
HYRX: Hyporoxia
